# Elementary Quantitive Analyses

**Published:** 1879-01

**Authors:** 


*Elementary Quantitive Analyses.* By ALEXANDER CLASSEN, Professor in the Royal Polytechnic School, Aux-La-Chapelle. Translated with additions, by EDGAR SMITH, A. M., Ph. D. With thirty-six illustrations. Philadelphia: Henry C. Lea, 1878. Buffalo: Theodore H. Butler.

We think this work will be regarded with great favor by the student of Chemistry, as it seems to us invaluable in the laboratory of the practical Chemist. It is elegantly illustrated, and all the processes of examination are fully and correctly explained. We earnestly recommend it to our readers.

